# Oral Allergy Syndrome: An Update for Stomatologists

**DOI:** 10.1155/2015/543928

**Published:** 2015-11-08

**Authors:** Roopashri Rajesh Kashyap, Rajesh Shanker Kashyap

**Affiliations:** ^1^Department of Oral Medicine and Radiology, A.J. Institute of Dental Sciences, Mangalore 575004, India; ^2^Department of Periodontics, Yenepoya Dental College, Mangalore 575018, India

## Abstract

Oral allergy syndrome (OAS) is an allergic reaction in the oral cavity subsequent to the consumption of food such as fruits, nuts, and vegetables. It occurs mainly due to homology of proteins of pollen to the proteins of fruits and vegetables. In OAS, the immune system produces antibodies that are directed against the proteins of pollen and structurally similar proteins in food, hence, resulting in allergic symptoms limited mainly to the oral cavity. In this review, we have summarized the etiopathogenesis, clinical features, diagnosis, and management of OAS as an update for stomatologists.

## 1. Introduction

Oral allergy syndrome (OAS) is an allergic reaction in the oral cavity subsequent to the consumption of food, such as fruits, nuts, and vegetables, which occurs in adults who suffer from allergic rhinitis [1]. It has been described under various names including “pollen-food allergy syndrome,” “pollen-food syndrome,” and “pollen-associated food allergy syndrome” [2]. OAS in adults probably represents the most common allergic reaction caused by food; and more than 60% of all food allergies are actually cross-reactions between food and inhaled allergens. Unlike other food allergies, OAS is a reaction limited to the oral mucosa, lips, tongue, and throat [1].

The first description of the OAS that associated a hypersensitivity to fruits and vegetables to birch pollinosis was written in 1942 by Tuft and Blumstein. Amlot et al. in 1987 first denominated it as “oral allergy syndrome” upon presenting a mainly oral clinical manifestation [3]. Due to the increasing popularity of exotic fruits and vegetables in the diet, an increasing number of pollen-allergic patients exhibit allergic reactions to these delicacies [4].

## 2. Etiopathogenesis

OAS manifestations occur after the patient who is allergic to pollen consumes certain fruits, vegetables, or nuts. OAS belongs to the allergy type I group, that is, allergic reactions mediated by immunoglobulin E (IgE). In susceptible patients, the immune system produces IgE antibodies against the proteins of pollen which causes hay allergy. Pollen allergies are caused by repeated exposure to the pollen of some plants, which are usually pollinated by air and have such pollen quantities that inhalation of the pollen easily reaches the surface of the pulmonary alveoli. The proteins which are structurally similar to pollen are also found in food. The OAS patient is first sensitized by inhaling pollen that contains the antigens, and then after consuming food that contains cross-antigens (to the inhaled antigens) the symptoms characteristic of OAS appear [1].

Plant-derived proteins responsible for allergy include various families of pathogenesis-related proteins, protease and *α*-amylase inhibitors, peroxidases, profilins, seed-storage proteins, thiol proteases, and lectins, whereas homologous animal proteins include muscle proteins, enzymes, and various serum proteins [5].

Cross-reactivity between birch pollen and various fruits and vegetables is due to homology among various pathogenesis-related proteins, which are important in the defense against plant diseases. For example, Mal d 1, the major apple allergen, is 63% homologous to the major birch pollen allergen, Bet v 1. Other birch pollen-related, pathogenesis-related proteins have been identified in hazelnut and celery (Api g 1). Similarly, the birch pollen profilin, Bet v 2, cross-reacts with profilins found in apple (Mal d 2), celery (Api g 2), and potato [6]. The same immune system can trigger allergic symptoms in two different ways: in the presence of pollen it leads to rhinitis and in the presence of a particular food it leads to symptoms of food allergy. Different allergens vary in their stability, with differences in digestion survival, storage, high temperature, cold, and cooking or pasteurization survival. As important drivers of anaphylaxis, the lipid transporting proteins play an important role, since they cannot be easily denatured by digestion or cooking. Antibodies can react to linear amino acid sequences of the protein or a conformational epitope. Persons who respond to the linear sequence of the protein can tolerate neither raw nor cooked food, while those that respond to a conformational epitope can consume cooked food but not the raw food [1].

Certain foods like peanuts are able to sensitize and elicit reactions after oral exposure and could trigger responses that generalize to related foods (legumes). In other groups of foods like apples, sensitization to homologous proteins encountered through respiratory exposure (e.g., birch pollen) may mediate reactions to cross-reacting proteins in the food [5].

IgE mediated food allergy is classified as classes 1 and 2. This distinction is based on clinical appearance, the predominantly affected group of patients (children or adults), and disease-eliciting food allergens. Primary (class 1) food allergy starts in early life and often represents the first manifestation of the atopic syndrome. The most common foods involved are cow's milk, hen's egg, legumes (peanuts and soybean), fish, shellfish, and wheat. The allergens contained in these foods not only elicit allergic reactions in the gastrointestinal tract but also often cause urticaria, atopic dermatitis, and bronchial obstruction. With a few exceptions, most children outgrow class 1 food allergy within the first 3 to 6 years of life [7].

Secondary (class 2) food allergy describes allergic reactions to foods in mainly adolescent and adult individuals with established respiratory allergy, for example, to the pollen of birch, mugwort, or ragweed. This form of food allergy is believed to be a consequence of immunological cross-reactivity between respiratory allergens and structurally related proteins in the respective foods. OAS belongs to this group [7].

Food allergens that induce OAS rapidly dissolve in the oral cavity and are readily broken down by digestive enzymes [8]. Preservatives in foods may also trigger the manifestation of the disease [1]. Due to the structural similarity of individual protein molecules, a large number of allergens that exist in nature can be classified into groups as follows [1, 8] (Table 1).

Among the allergens in each group there is a possibility of cross-reactivity of IgE antibodies, that is, antibodies binding to one of two or more allergens. The reaction may start with one type of food, and subsequently allergies to other food types can develop [1].

Latex allergens can also sensitize patients to cross-react to the protein found in some foods [2]. One of the most notable features of latex allergy is the patients' cross-reactivity to various fruits and vegetables, a condition often called* latex-fruit syndrome*. The first report of an allergic reaction to banana in a latex allergic patient was published in 1991 [9, 10]. Structurally similar proteins in many kinds of plants must be responsible for such extensive cross-reactivity [11]. Commonly reported cross-reactive foods include banana, avocado, kiwi, chestnut, potato, and papaya, and numerous latex allergens cross-react with food and pollen proteins [5].

Rarely OAS is induced by the ingestion of other foods in subjects without pollen sensitization, for example, shellfish and pork [12, 13]. Honey is considered to be another food which can cause OAS. During collection, grains of pollen are admixed to this raw material which retains their allergenic properties during the honey making process [14].

More and coworkers reported that some patients presented with OAS symptoms after eating foods cooked over mesquite wood and individuals were positive to skin prick test with mesquite pollen extract. They concluded that the transfer of allergens in foods cooked over mesquite wood might lead to symptoms in sensitized individuals [15].

## 3. Clinical Features

OAS symptoms may vary from person to person. Some studies have confirmed that the OAS is more common in female patients [16, 17]. Patients who show symptoms of OAS may have a number of other allergic reactions that start very quickly, even minutes after consuming trigger food. Usually, it is manifested by itching and a burning sensation of the lips, mouth, ear, and throat or by the appearance of perioral erythema and generalized urticaria. Sometimes, reactions can manifest themselves in the eyes, nose, and skin. The patient may develop swelling of the lips, tongue, and uvula, occasionally a sense of suffocation, and rarely anaphylaxis. Symptoms usually last for a few minutes to half an hour. In rare situations, OAS may be manifested as difficulty in breathing, appearance of a rash, or hypotension [1] (Table 2).

Pastorello et al. have recorded symptoms after oral challenge with offending food and the reaction was classified in 4 grades of severity: (I) only oral mucosa symptoms; (II) oral mucosa and gastrointestinal symptoms; (III) oral mucosa and systemic symptoms, such as urticaria, angioedema, rhinoconjunctivitis, and asthma; and (IV) oral mucosa and life-threatening symptoms, such as laryngeal edema and shock [18]. However, in most of the cases, OAS presents with mild symptoms.

## 4. Diagnosis

Food allergy diagnostics are one of the most difficult tasks in allergology, especially when there is no clear connection between the development of the clinical features and the ingested food or when food allergy takes an atypical or chronic course. The diagnostic methods can be divided into two groups: clinical and laboratory. Among the group of the clinical methods clinical history, eating habits investigation, skin tests, and challenge tests are used for their high informative value. Trial elimination diets are also applied. The specific IgE antibodies assay is the most important among the laboratory methods [19].

For a correct diagnosis, it is necessary to obtain a thorough patient history. A diagnosis of OAS is based primarily on clinical history. History of an allergy, whenever reported, should be recorded in the patient's medical history [1, 20]. Clinical history should contain details about the development of the clinical features, food eaten, symptoms, and period of time between the intake of the food and the onset of the signs, and sequence of the manifestations [19]. In patients with allergies to airborne particles, the appearance of oral itching or tingling after eating fresh fruit or vegetables is enough to suspect OAS [3].

Skin testing for IgE mediated reaction can be carried out using different methods: the prick method (prick test), the application of allergens* via* scratching the skin (scratch test), and rarely an intradermal test (application of allergens into the skin by a needle) [1]. Commercial extracts are used for prick tests, determining allergy to peanuts, hazelnuts, and peas. Prick tests are not carried out in areas of dermatitis or in areas where dermocorticosteroids or immunomodulating creams have been applied [19].

The skin prick test is performed with commercial extracts of pollens and food on the forearm or the back, measuring the wheal after 15 minutes, and is considered positive if the diameter of the wheal is greater than 2 mm of the negative control sample [3]. The commercially available fruit extracts used in allergy testing are not usually reliable indicators of allergy in patients with oral allergy syndrome, because the cross-reactive epitopes have been destroyed by the manufacturing process. Prick-plus-prick testing (prick the fruit and then prick the skin) with freshly prepared fruit extracts is more sensitive in detecting allergen specific IgE antibody [20].

If the history is positive and the prick test is negative, a provocation test with a fresh food should be conducted. An oral provocation test represents the safest confirmation of the presence of the disease. In doing so, the person first consumes a suspected food, and subsequently the onset of symptoms is recorded. To set up an accurate diagnosis, it would be necessary to keep a diary of food consumption as the basis for determination of which food tests to undertake. Good history can focus the testing on a specific type of food, and thus the doctor can act more rationally [1]. For most IgE mediated reactions, 8 ± 10 gm of the dry food or 100 mL of wet food (double amount for meat/fish) at 10 ± 15 min intervals is given over about 90 min followed by a larger, meal size portion of food a few hours later. The symptoms should be recorded and frequent assessments are to be made for symptoms affecting the skin, gastrointestinal tract, and/or respiratory tract [21].

Blood tests are mostly performed as RIST (Radioimmunosorbent Test) for the determination of total IgE and RAST (Radioallergosorbent Test) for the determination of specific IgE antibodies to a particular allergen. A blood test is usually used when there is no possibility of skin tests [20].

Extensive research has led to the identification of principle allergens in cross-reactive food. Many allergenic components have been produced in a recombinant form maintaining their immunoreactivity and allergenic epitopes. These allergens are applied to a chip based microarray that uses small quantities of serum and provides IgE antibody profiles to over 100 food and pollen allergens. However, most of the in vitro diagnostic tests are expensive and the cost factor limits its use [22].

## 5. Management

A multidisciplinary approach in patients with OAS is necessary, which involves different professions (ear-nose-throat specialists, oral pathologists, allergologists, immunologists, dermatologists, pediatricians, gastroenterologists, and various other specialties) [1]. There is no standard treatment established for OAS except avoiding implicated food [23].

The OAS should be managed according to the clinical presentation [20]. Since many of the immunogenic proteins in fruits and/or vegetables are unstable (heat-labile), patients will tolerate food cooked and canned well and fresh or raw foods badly [3]. It has been shown that cooking food can sometimes eliminate allergens in certain species like apples, while it is impossible to destroy allergens in celery and strawberries. For some types of food (e.g., nuts) that contain more than one allergen, heat treatment will destroy certain allergens, while some of them can cause a reaction even after that [1].

Education is the key pillar of an effective long-term elimination diet. Patients, their families, close relatives, and caregivers should be aware of risk situations and should be instructed in reading labels and how to avoid the relevant food allergens both inside and outside the home [24]. Most patients with OAS can be treated with a combination of allergen avoidance and pharmacotherapy. The most important therapy includes antihistamines. Oral antihistamines such as cetirizine 10 mg [25] or intramuscular aqueous epinephrine at the dose of 0.01 mL/kg of 1 : 1000 dilution can alleviate allergic symptoms by blocking specific immune pathways [26]. The use of topical preparation of mast cell stabilizers like cromolyn sodium or antihistamines like levocetirizine prior to food intake has been effective in helping some patients with food allergy. This medication works by blocking mast cell mediator release [27]. Patients with a history of anaphylaxis should always carry a shot with a dose of epinephrine (such as EpiPen which contains epinephrine 0.3 mg in 0.3 mL) with them [28].

In the event of a reaction, the patient is advised to stay calm, rinse their mouth with plain water, and rest. The patient can help himself/herself with hot (but not boiling) beverages that can inactivate residual allergens. This usually leads to withdrawal of the sensation of prickling, itching, and swelling, which stops within 30 minutes to an hour (before the antihistamine makes an effect) [1, 29]. When the patient is able to swallow a dose of antihistamines, they definitely need to be taken. However, severe symptoms are rare in patients with OAS [1, 29].

In patients with suspected OAS, preemptive caution is necessary because the preparation of food can be connected to reactions. Different reactions may appear at different times, such as sneezing attacks during scraping of fruits and vegetables, when particles can get into the air, or conjunctivitis if the patient touches his/her eyes after touching the fruit or vegetables. Wearing gloves and masks can help prevent contact with allergens. It is also recommended to avoid latex (rubber gloves) that can cause cross-allergic reactions to foods of plant origin. If the patient avoids areas of certain types of pollen, the syndrome usually relieves after two to three years [1].

Desensitization to the pollen with immunotherapy is recommended in some cases and can sometimes help minimize cross-reactions [2]. Immunotherapy may be beneficial if a single allergen is implicated [20]. Subcutaneous specific immunotherapy (SIT) has been tried and has significantly reduced OAS symptoms associated with ingestion of the responsible fruit and vegetables [22]. According to a study by Asero, at least in some patients pollen SIT can exert a long-lasting effect on pollen-associated food allergies (patients sensitized to birch pollen were still able to eat apples without any complaints as long as 30 months after the end of SIT) [30]. A study by Bergmann et al. also suggested that pollen-specific SIT can reduce OAS triggered by pollen-associated foods in patients with pollen-induced rhinoconjunctivitis [31]. Further researches are still going on on immunotherapy as a treatment modality for OAS.

## 6. Conclusion

Allergy or intolerance to the food we eat may be a problem routinely encountered. Though OAS is mainly managed in allergy clinics, it is equally important for the oral physicians to be aware of the symptoms and clinical features of OAS. It is equally important to record patient's history accurately regarding previous episodes of allergies. An orderly approach should be followed in managing the patients with OAS. The dentists should pay attention especially to the individuals with a history of asthma, atopy, or any other allergic problems during the dental treatment procedures. Even though the symptoms of OAS are mild in most of the cases, they can manifest life-threatening complications occasionally.

## Figures and Tables

**Table 1:** Types of pollen and food associated with oral allergy syndrome.

Pollen	Fruit	Vegetable	Nuts	Grains
Birch	Kiwi, apple, pear, plum, peach, nectarine, apricot, cherry, banana, fig, avocado, strawberry, dried plum, mango	Celery, carrot, parsnip, parsley, dill, cumin, cilantro, fennel, potato, tomato, pepper (green), chicory	Hazelnut, almond, walnut, peanut	Soybeans, wheat, lentils, peas, beans

Ragweed	Banana, watermelon, melon, honey dew, cantaloupe	Squash, pepper, cucumber, artichoke, hibiscus, zucchini, chamomile tea		Sunflower seeds

Weeds	Melon, watermelon, orange, kiwi	Tomato		

Wormwood	Apple, watermelon, melon	Celery, carrot, parsley, pepper, cilantro, fennel		

*Parietaria *	Cherry, melon			

Grass	Fig, melon, orange, kiwi, watermelon	Tomato, potato	Peanut	

*Alder *	Apple, cherry, peach, pear, strawberry, raspberry	Celery, parsley	Hazelnut, almond, walnut	

Japanese cedar		Tomato		

Mugwort	Mango	Celery, carrot		

Plane	Apple	Lettuce, corn	Hazelnut, peanut	Chickpea

**Table 2:** List of reported cases on various food items causing OAS symptoms.

S. number	Food	Manifestation	Reference
1	Jackfruit	Itching and burning in the mouth and throat	[4]
2	Salami	Mild pruritus in oral mucosa	[12]
3	Pork	Itching and angioedema of the lips and oral mucosa	[13]
4	Honey	Itching in the mouth, gastrointestinal symptoms, and angioedema	[14]
5	Tomato juice	Dyspnea, swelling of the oral and nasal mucosa, and congestion of the bulbar conjunctiva	[32]
6	Raw fish	Oropharyngeal irritation and facial angioedema	[33]
7	Peanut	Lip tingling, oral itching, lip swelling, and throat itching	[34]
8	Cashew nut	Sialorrhea, perioral urticarial rash, tongue swelling, and immediate vomiting	[35]
9	Grapes	Flushing of the face and neck, followed by local itchy skin rash, itching and edema of the oral and perioral mucosa, and moderate dyspnea	[36]
10	Sapodilla plum	Lip edema, accompanied by itching in the lips, tongue, and throat, as well as a feeling of dryness and hardness in the throat (glottic edema)	[37]
11	Cooked aubergine	Oral itching and significant perioral urticaria	[38]
12	Pistachio nuts	Oral and lip itching and swelling	[39]
13	Royal jelly	Lip, tongue edema, and palate itching	[40]
14	Mango	Oropharyngeal itching, tiredness, dizziness, and swelling of the face	[41]
